# Cellular basis of enhanced humoral immunity to SARS-CoV-2 upon homologous or heterologous booster vaccination analyzed by single-cell immune profiling

**DOI:** 10.1038/s41421-022-00480-5

**Published:** 2022-10-21

**Authors:** Jingwen Ai, Jingxin Guo, Haocheng Zhang, Yi Zhang, Haochen Yang, Ke Lin, Jieyu Song, Zhangfan Fu, Mingxiang Fan, Qiran Zhang, Hongyu Wang, Yuanhan Zhao, Zhangyufan He, An Cui, Yang Zhou, Jing Wu, Mingzhe Zhou, Guanmin Yuan, Boxi Kang, Ning Zhao, Yuanyuan Xu, Mengqi Zhu, Youhong Wang, Zemin Zhang, Ning Jiang, Chao Qiu, Chenqi Xu, Wenhong Zhang

**Affiliations:** 1 National Medical Center for Infectious Diseases, Huashan Hospital, Fudan University, Shanghai, China; 2 Department of Infectious Diseases, Shanghai Key Laboratory of Infectious Diseases and Biosafety Emergency Response, Huashan Hospital, Fudan University, Shanghai, China; 3 State Key Laboratory of Molecular Biology, Shanghai Key Laboratory of Molecular Andrology, Shanghai Institute of Biochemistry and Cell Biology, Center for Excellence in Molecular Cell Science, Chinese Academy of Sciences, Shanghai, China; 4 University of Chinese Academy of Sciences, Beijing, China; 5 Department of Urology, Tongji Hospital, School of Medicine, Tongji University, Shanghai, China; 6 Analytical Biosciences Limited, Beijing, China; 7 Biomedical Pioneering Innovation Center (BIOPIC), School of Life Sciences, Peking University, Beijing, China; 8 State Key Laboratory of Genetic Engineering, School of Life Sciences, Fudan University, Shanghai, China; 9 Institutes of Biomedical Sciences, Fudan University, Shanghai, China; 10 School of Life Science and Technology, ShanghaiTech University, Shanghai, China; 11 Key Laboratory of Systems Health Science of Zhejiang Province, School of Life Science, Hangzhou Institute for Advanced Study, University of Chinese Academy of Sciences, Hangzhou, Zhejiang China; 12 National Clinical Research Center for Aging and Medicine, Huashan Hospital, Fudan University, Shanghai, China; 13 Huashen Institute of Microbes and Infections, Shanghai, China

**Keywords:** Transcriptomics, Gene expression profiling, Bioinformatics

## Abstract

SARS-CoV-2 vaccine booster dose can induce a robust humoral immune response, however, its cellular mechanisms remain elusive. Here, we investigated the durability of antibody responses and single-cell immune profiles following booster dose immunization, longitudinally over 6 months, in recipients of a homologous BBIBP-CorV/BBIBP-CorV or a heterologous BBIBP-CorV/ZF2001 regimen. The production of neutralizing antibodies was dramatically enhanced by both booster regimens, and the antibodies could last at least six months. The heterologous booster induced a faster and more robust plasmablast response, characterized by activation of plasma cells than the homologous booster. The response was attributed to recall of memory B cells and the de novo activation of B cells. Expanded B cell clones upon booster dose vaccination could persist for months, and their B cell receptors displayed accumulated mutations. The production of antibody was positively correlated with antigen presentation by conventional dendritic cells (cDCs), which provides support for B cell maturation through activation and development of follicular helper T (Tfh) cells. The proper activation of cDC/Tfh/B cells was likely fueled by active energy metabolism, and glutaminolysis might also play a general role in promoting humoral immunity. Our study unveils the cellular mechanisms of booster-induced memory/adaptive humoral immunity and suggests potential strategies to optimize vaccine efficacy and durability in future iterations.

## Introduction

COVID-19 (coronavirus disease 2019) has been detected in more than half a billion cases and linked to over six million deaths reported to the World Health Organization (WHO)^1^, control of which remains a global priority. Vaccination is the cornerstone of pandemic control. Globally, nearly billions of doses of various COVID-19 vaccines have been administered. Clinical evaluation of the vaccine-induced immune response, which usually involves immunogenicity, safety, and protection efficacy, could aid the decision-making on public-health strategy. Among the different vaccines developed during the pandemic, inactivated vaccines are currently widely used in China and other regions of the world. Protein subunit COVID-19 vaccines, using the receptor-binding domain (RBD) of the spike protein as antigens, require less stringent cold-chain logistics and storage, a factor that facilitates vaccine accessibility in the global supply^2^.

Previous studies have found that vaccinated cases have improved clinical outcomes^3^. However, waning vaccine effectiveness has been observed against COVID-19-related hospitalization and death 5–7 months after the second dose of the primary series, especially for various immune-evasive variants. A booster dose of either homologous or heterologous vaccine appears to increase the protectiveness against hospitalization and to prevent disease progression into severe stages^4^. The adjusted vaccine effectiveness against symptomatic COVID-19, based on a real-world study, was estimated to be 78.8% with three doses of inactivated vaccine and 93.2%–96.5% for a heterologous booster^5^. Such adjusted vaccine effectiveness rates were 86.3% against hospitalization and 86.7% against COVID-19-associated deaths following a three-dose inactive vaccine schedule^5^. Heterologous boosters seemed to show higher vaccine effectiveness than homologous boosters for all evaluated clinical endpoints.

After vaccination, the immune system retains a memory ability which provides protection from subsequent infection and prevents disease progression into the severe stage. Memory cells of the adaptive immune system and antibodies that patrol in the body can recognize the invader and generate a swift response upon re-encountering. The period that these components last in the body determines the durability of immune memories. In most studies, these are quantified by the titers and spectrum of antibodies and the magnitude of antigen-specific B cells and T cells. Waning antibody titer occurs after the primary vaccine series and thus a booster dose is advocated. However, the durability of the antibodies to previously and currently circulating variants following the booster dose, the exact cellular process of booster-activated B cell immunity and how long memory B cells could persist are opaque.

Massive single-cell 5’ mRNA and V(D)J sequencing (scRNA/V(D)J-seq) could provide a landscape view of the cellular heterogeneity and immune repertoire diversity at single-cell resolution. Previously, this approach has been employed in demonstration of the BNT162b2 vaccine-induced antigen-specific CD8 T cell responses^6,7^. Collectively, scRNA-seq and scV(D)J-seq could help understand the B and T cell clonality, vaccine-induced cellular phenotypes and transcriptional signatures, which could greatly assist in the intervention of COVID-19.

Herein, we investigated both the activation and memory phases of adaptive humoral immune responses following a booster dose of RBD-subunit vaccine (ZF2001) and inactivated vaccine (BBIBP-CorV), primed with two-dose inactivated vaccines. Taking advantage of single-cell immune profiling, we unveil the cellular basis for the boosting effect and highlight key metabolic pathways relevant to antibody production, both of which may lead the development of a next-generation SARS-CoV-2 vaccine with higher and more durable efficacy.

## Results

### Durable response of neutralizing antibodies induced by a heterologous or homologous booster dose

**Fig. 1:**
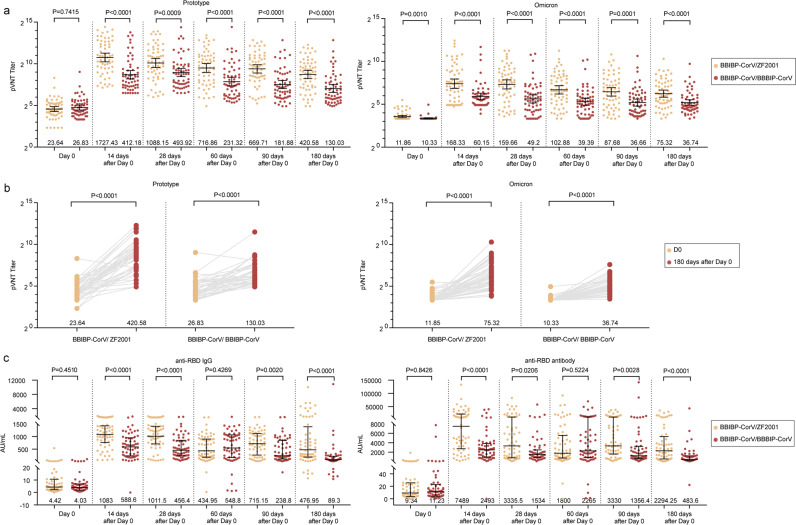
Longitudinal dynamics of neutralizing antibody responses induced by the booster vaccines. **a** Neutralizing antibody titers after the third-dose vaccination against the prototype (left) or Omicron variant (right) evaluated by pVNT. The horizontal bars indicate the GMT, and error bars indicate the 95% confidence interval. **b** Neutralizing antibody titers against the prototype (left) or Omicron variant (right) in BBIBP-CorV/ZF2001 heterologous booster group and BBIBP-CorV/BBIBP-CorV homologous booster group. Neutralizing antibody titer against the Omicron variant in BBIBP-CorV/ZF2001 heterologous booster group and BBIBP-CorV/BBIBP-CorV homologous booster group. **c** Concentrations of anti-RBD IgG (left) or anti-RBD antibody (right) against the prototype after the third-dose vaccination. The horizontal bars indicate the median concentration, and error bars indicate the inter-quartile ranges.

We carried out a pseudovirus neutralization test (pVNT) of all the enrolled recipients and evaluated the neutralizing titer post-homologous BBIBP-CorV/BBIBP-CorV or post-heterologous BBIBP-CorV/ZF2001 booster vaccination (Fig. 1a). The heterologous group showed substantially higher pVNT values than those of the homologous group during the 6-month follow-up period. Although the neutralizing titers against the Omicron BA.1 variant were lower than those against the prototype strain, it was still retained for at least 6 months.

The Omicron pVNT level 180 days (GMT 75.32, 95% CI: 55.13–97.39) after the booster dose in the BBIBP-CorV/ZF2001 group was 6.35 times higher than the baseline (GMT 11.86, 10.87–13.02), while the level in the BBIBP-CorV/BBIBP-CorV group (GMT 36.74, 29.32–46.06) was 3.56 times higher than the baseline (GMT 10.33, 9.91–10.76, *P* < 0.0001). The tendency of pVNT titer against the prototype strain was similar to that against the Omicron Variant within 180 days post booster dose (Fig. 1b). The levels of anti-RBD total antibody and IgG isotype were also assessed during follow-up (Fig. 1c). Consistently, both boosters induced long-term production of anti-RBD antibodies, showing the similar trajectory as the neutralizing titer level. Moreover, the heterologous group displayed higher antibody levels than the homologous group.

We next compared antibody waning 6 months post vaccination against other published datasets (Supplementary Fig. S1a). For the three-dose BBIBP-CorV/ZF2001 and BBIBP-CorV groups in this study, 44.04% and 15.17% of the peak neutralizing antibody activity were retained at 6 months. For the three-dose CoronaVac and BNT162b2 groups, 27.20% and 27.77% were retained^8,9^. For the two-dose CoronaVac and BNT162b2 groups, antibody waning was more evident, showing only 9.51%, and 4.69%–10.11% of peak levels^8,10–12^. Thus, the heterologous booster strategy displayed certain advantage in inducing sustainable humoral immunity.

### Global profiling of peripheral blood mononuclear cells

**Fig. 2:**
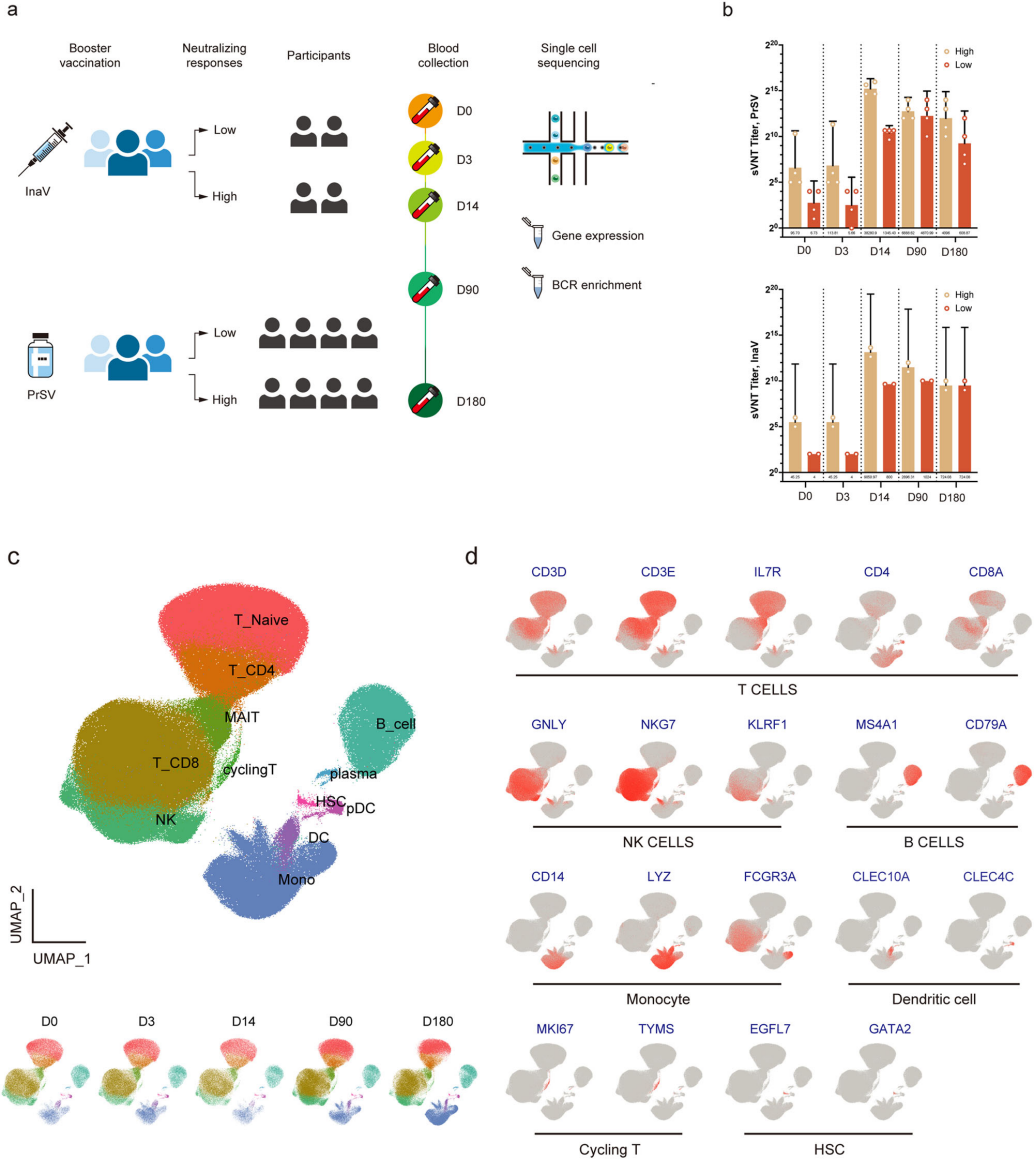
Profiling of PBMCs by scRNA-seq and scV(D)J-seq. **a** Schematic diagram showing the experimental design for scRNA-seq and scV(D)J-seq. PBMCs were collected from 12 participants. **b** Humoral immune responses (sVNT) of each sample at Day0, Day3, Day14, Day90 and Day180 in PrSV group (top) and InaV group (bottom), respectively. **c** UMAP representation of PBMCs from all participants and time points sampled (top), and UMAPs annotated by time point separately (bottom). Cell types were identified by cluster gene signatures and color-coded. Each dot represents an individual cell. **d** UMAP projection of canonical markers of each cell type.

Next, we used scRNA-seq and scV(D)J-seq to study the dynamics of immune cell profiles and B cell clones post homologous and heterologous booster vaccination. Since recipients displayed large variations during each booster dose, we selected those with extremely high or extremely low antibody titers to study the cellular basis for antibody response (Supplementary Table S1). In the homologous group (InaV), we selected two subjects who were in the top quartile of neutralizing antibody titer (InaV H group), and two subjects in the bottom quartile by Day 3 (D3) and D14 (InaV L group). In the heterologous group (PrSV), four subjects in the top quartile (PrSV H group) and four subjects in the bottom quartile (PrSV L group) were enrolled (Fig. 2a).

The surrogate virus neutralization test (sVNT) was carried out at D0, D3, D14, D90, and D180 post booster in the scRNA-seq group (Supplementary Fig. S1b). We found that B cell immunity was promptly activated within two weeks of vaccination, declining gradually thereafter. The sVNT titer of the heterologous booster group was overall higher than that of the homologous group during the six months of follow-up. On D14, the pVNT titers in the PrSV H and PrSV L groups peaked at 38,280 and 1345, respectively, representing ~401 and 199 times of D0 in each group. On D180, the antibody levels in the PrSV H and PrSV L groups decreased to 4096 and 608, respectively. However, the neutralizing activity was retained in both high- and low-antibody-titer groups. The same phenomenon was observed in the homologous booster group. Six months after vaccination, the PrSV H group had a 5.66-fold higher antibody level than the InaV H group (Fig. 2b).

Peripheral blood mononuclear cells (PBMCs) were collected prior to (D0) and on D3, D14, D90, and D180 after the booster vaccination (Fig. 2a). After quality filtering, we obtained a total of 454,202 single-cell transcriptomes, with a median expressed gene and unique molecular identifier (UMI) counts of 1465 and 3845, respectively. We applied Uniform Manifold Approximation and Projection (UMAP) for visualization and clustered cells using a graph-based method, which yielded 12 clusters (Fig. 2c). In particular, we identified six major known cell types by their unique signature genes, including B cells (*CD19, CD79A, CD79B*), T cells (*CD3D, CD4, CD8A, KLRB1*), dendritic cells (DCs) (*S100A9, CLEC10A*), monocytes (*CD14, FCGR3A/CD16*), natural killer (NK) cells (*NKG7, TRDC, NCAM1*), and hemopoietic stem cells^13^ (*EGFL7, GATA2*) (Fig. 2d). In addition to the transcriptome analysis, we also used single-cell B cell receptor (BCR) sequencing (scBCR-seq) to investigate clonal expansion in the activation phase and persistence in the memory phase of B cell immunity.

### Heterologous booster induced more robust activation of plasma cells than homologous booster

**Fig. 3:**
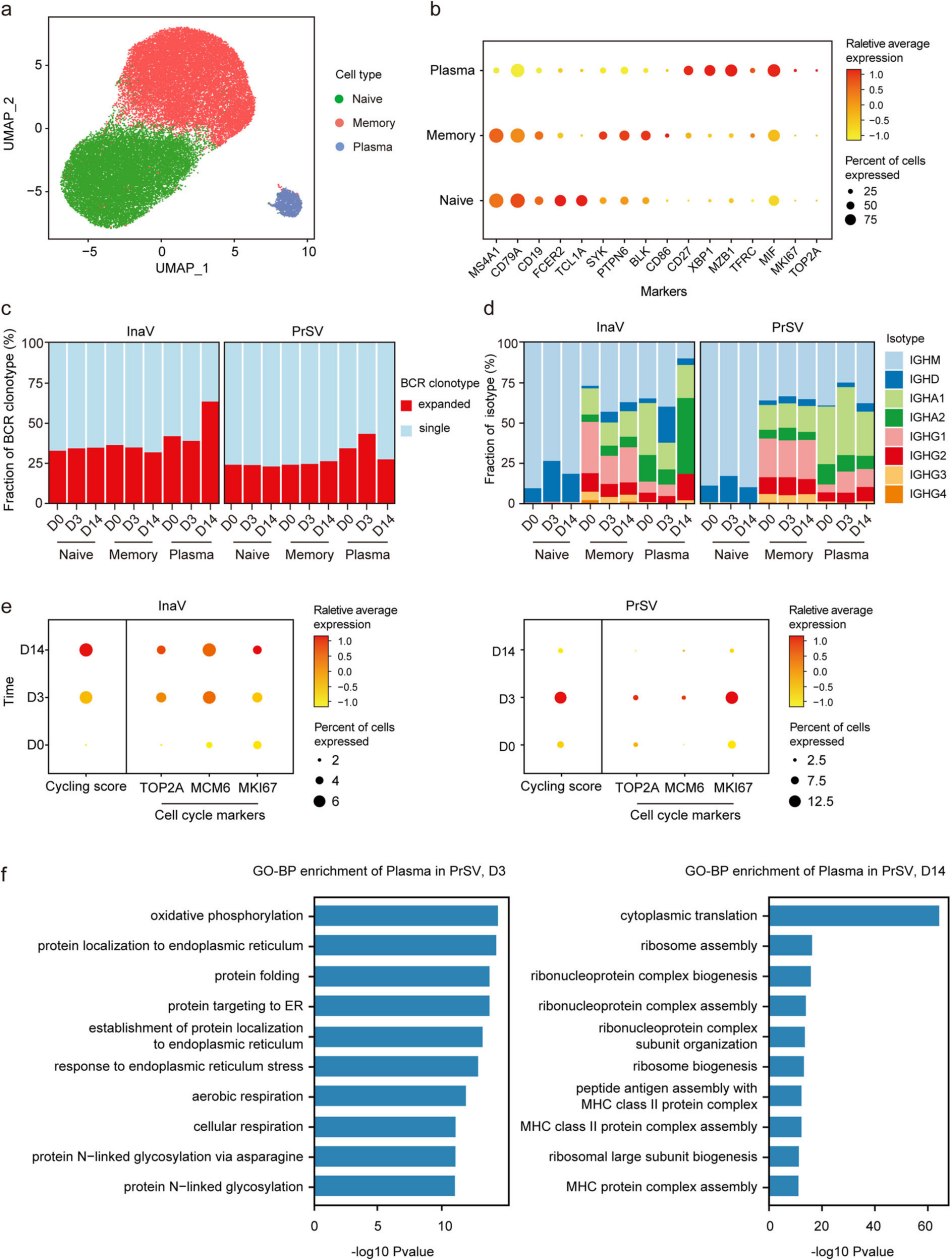
Acute B cell activation following by the booster vaccination. **a** UMAP representation of B cells derived from PBMCs. Clusters are denoted by color and labeled with inferred cell types. **b** Dot plot of average expression and percentage of expressed cells of selected canonical markers in each labeled B cell subtype. **c** BCR clone differences across time points in each B cell subtype at D0, D3, and D14. **d** The relative percentage of each isotype in each B cell subtype at D0, D3, and D14. **e** Dot plot shows the cell cycle score and average expression of cell cycle markers, *TOP2A, MCM6* and *MKI67*, in plasma cells. Dot size depicts the percentage of the indicated gene-expressing cells. **f** The enriched GO-BP terms of upregulated genes in plasma cells in PrSV group at D3 (left) or D14 (right) post-vaccination. *P* value was derived by Benjamini & Hochberg test.

Within the B cell population, we identified three major subtypes, i.e., naïve B cells (*MS4A1, IGHD, TCL1A*), memory B cells (*MS4A1, CD27, PTPN6, BLK*) and plasma cells (*XBP1, MZB1*) (Fig. 3a, b). Plasma cells (plasmablast and plasma cell), as the major source of antibody secreting, displayed fast clonal expansion in response to booster vaccination (Fig. 3c). The heterologous group showed earlier expansion dynamics than the homologous group. The major BCR isotypes of expanded clones were IGHA2 and IGHG2 in the homologous group and IGHA1 and IGHG1 in the heterologous group (Fig. 3d), revealing the qualitative difference in humoral immunity induced by different boosters (Fig. 1a–c). Consistent with the clonal expansion, plasma cell proliferation appeared earlier in the heterologous group (Fig. 3e). According to the gene ontology (GO) enrichment analysis, plasma cells in the heterologous group showed higher expression of genes associated with protein translation, folding and glycosylation. Moreover, oxidative phosphorylation in the mitochondria was more apparent (Fig. 3f). Comparing the differentially expressed genes (DEGs) of plasma cells at D3/D14 between PrSV and InaV groups, plasma cells from the PrSV group highly expressed genes involved in energy metabolism pathways like OXPHOS and aerobic respiration, indicating that plasma cells in the PrSV group were more active in producing and secreting antibodies after booster vaccination (Supplementary Fig. S2a, b). These features could partially explain the higher antibody production in the heterologous group.

### Early expansion and persistence of B cell clones underlies sustainable antibody titers

**Fig. 4:**
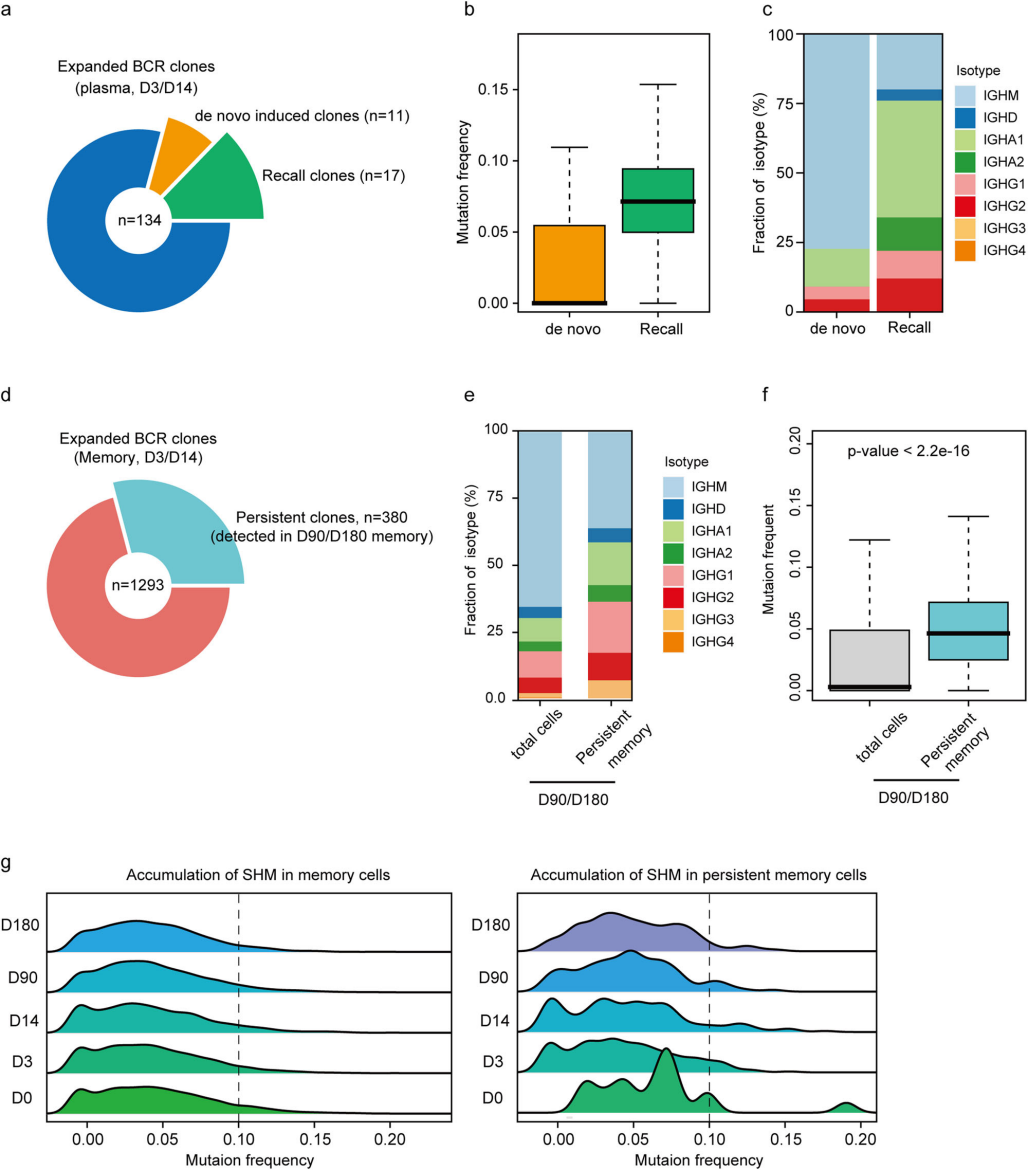
Clonal expansion and persistence of B cells. **a** Pie plot shows that 11/134 expanded BCR clones detected in D3/D14 plasma cells were derived from naïve B cell, named de novo induced clones, and 17/134 expanded BCR clones detected in D3/D14 plasma cells were derived from memory B cell, named recalled clones. **b** Boxplot shows the BCR mutation frequency of de novo induced clones and recalled clones. **c** Average proportions of BCR isotypes between de novo induced clones and recalled clones. **d** Pie plot shows that 380/1293 expanded BCR clones detected in D3/D14 memory B cells could be detected in D90/D180 memory B cells, named persistent clones. **e** Average proportions of isotypes of persistent clones compared with BCR clones detected at D90/D180. **f** Boxplot shows the BCR mutation frequency of persistent clones compared with BCR clones detected at D90/D180. **g** RidgePlot shows the accumulation of memory cells against booster vaccination.

To get deeper insights into the evolution of the adaptive humeral immune response against SARS-CoV-2 vaccines, we analyzed the overlap of BCR clones among B cell subtypes at different time points. To study the origin of B cells in response to the booster vaccination, we calculated the BCR clones shared between expanded clones of plasma cells at D3/D14 and total memory B cell clones detected at D0/D3/D14 (Fig. 4a). We found that 134 expanded clones detected in plasma cells were vaccination-related clones, and 17 of them had shared BCR with a subset of memory B cells, indicating the recall of SARS-CoV-2-specific memory B cells. Interestingly, another 11 expanded plasma cell clones had shared BCR with naïve B cells at D0/D3, suggesting de novo activation and differentiation of B cells in response to booster vaccination. Compared with the de novo clones, the recalled clones displayed higher frequencies of somatic hypermutation (SHM) (Fig. 4b) and isotype switching (Fig. 4c), suggesting that antibodies produced by the recalled clones might have better activity and breadth.

Next, we studied whether B cell memory induced by the booster could persist for a long period in the memory phase. Of the 1293 expanded memory BCR clones detected at D3/D14, 380 of them were detected at D90/D180, showing good persistence of B cell memory (Fig. 4d). Compared with total BCR clones detected at D90/D180, ~60% of these shared clones expressed IgA or IgG (Fig. 4e), in agreement with their origin of memory cells. In addition, these persistent clones showed higher SHM frequency than total clones (Fig. 4f). The SHM frequencies of persistent clones at D180 were even higher than those at D90 (Fig. 4g), suggesting continuous antibody affinity maturation.

In addition, we analyzed the differences of BCR clone types between InaV and PrSV groups. We found 10/17 recalled clones, 9/11 de novo clones and 159/380 persistent clones in the InaV group, as well as 15/17 recalled clones, 10/11 de novo clones and 233/380 persistent clones in the PrSV group. Thus, we surmise that both heterologous and homologous boosters can induce the antigen-related B cell activation and differentiation, and that the presence of SARS-CoV-2 antigen-specific BCR clones persists up to at least 180 days.

### High antibody titers are associated with active processes of antigen presentation and follicular helper T cell activation

Different recipients exhibited highly variable antibody titers in response to either homologous or heterologous booster vaccination. It is thus important to understand the immunological basis responsible for the variation. We re-grouped the homologous and heterologous booster recipients to high-antibody-titer and low-antibody-titer groups, analyzing the key immunological pathways regulating antibody responses, i.e., antigen presentation and follicular helper T-cell activation.

**Fig. 5:**
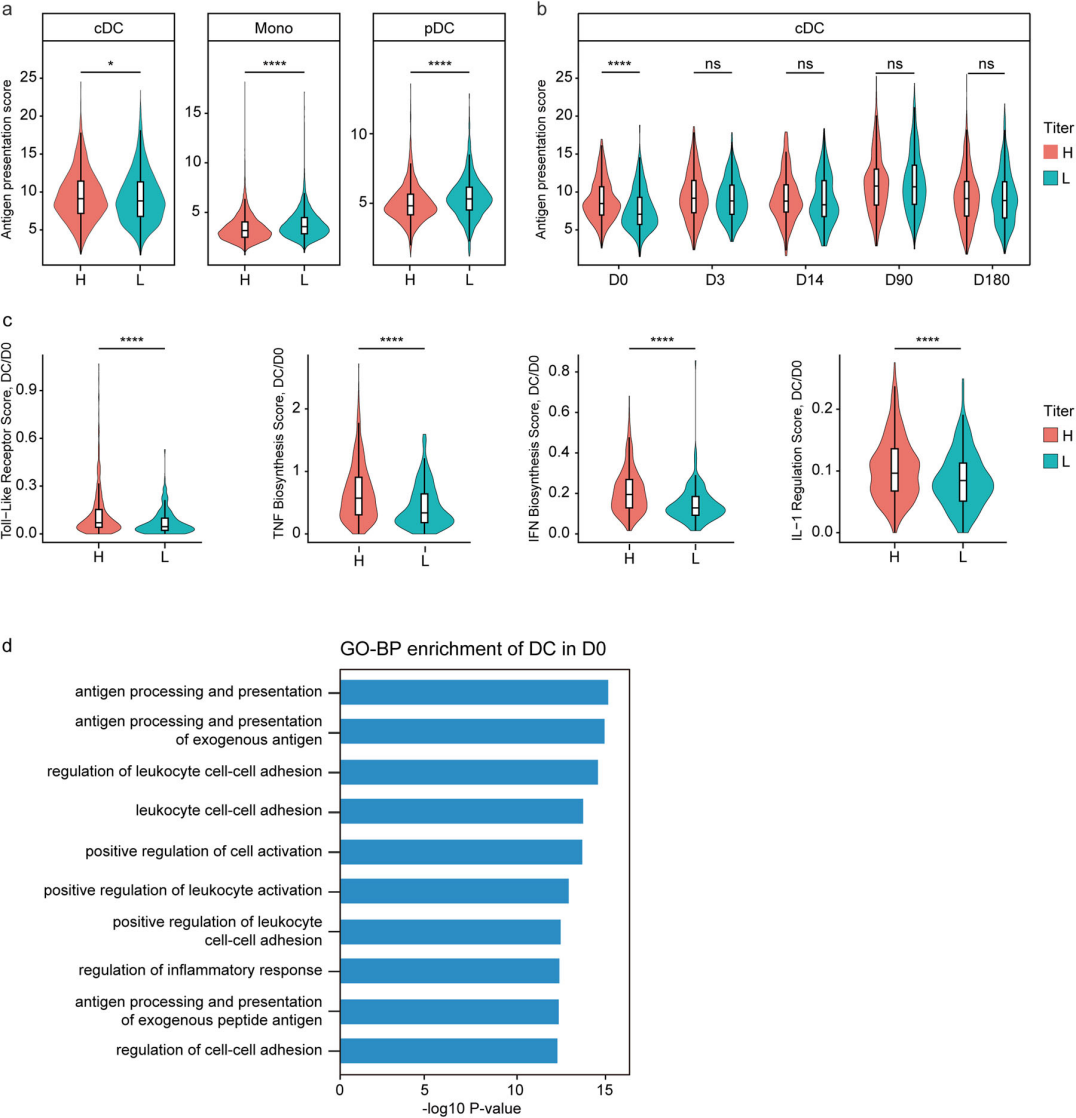
Conventional DCs are critical for antibody production. **a** Violin plots show the antigen presentation score of cDC, pDC cells and monocytes (Mono) between high- and low-titer groups. Values are means ± SD. *P < 0.05, **P < 0.01, ***P < 0.001. **b** Violin plots show the antigen presentation score of cDC between high- and low-titer groups at D0, D3, D14, D90 and D180, respectively. Values are means ± SD. *P < 0.05, **P < 0.01, ***P < 0.001. **c** Violin plots show Toll-like Receptor Score, TNF Biosynthesis Score, IFN Biosynthesis Score and IL-1 Regulation Score of cDC derived from D0 between high- and low-titer groups. Values are means ± SD. *P < 0.05, **P < 0.01, ***P < 0.001. **d** The enriched GO-BP terms of upregulated genes of cDC in high-antibody-titer group at D0. *P* value was derived by Benjamini & Hochberg test. The average of gene scores in **a**, **b** and **c** were showed in Supplementary Table S2.

We observed the professional antigen-presenting cells and found that antigen presentation activity of conventional dendritic cells (cDCs) showed positive correlation with antibody titers (Fig. 5a). In contrast, activity of plasmacytoid dendritic cells (pDCs) or monocytes displayed negative correlation with antibody titers. Further analysis of each time point showed that the correlation of cDC antigen presentation and antibody titer was only evident at D0 (Fig. 5b), suggesting that the basal cDC function plays a key role. In addition, Toll-like receptor signaling and cytokine signaling (TNF, IFN, and IL-1) were more active in cDCs in the high-antibody-titer group at D0, which could lead to improved cDC activation (Fig. 5c). GO-BP enrichment analysis supported that cDCs in the high-antibody-titer group had better cell activation, cell adhesion and antigen presentation (Fig. 5d). Thus, basal cDC activation and function may be crucial to triggering the booster-induced antibody response.

**Fig. 6:**
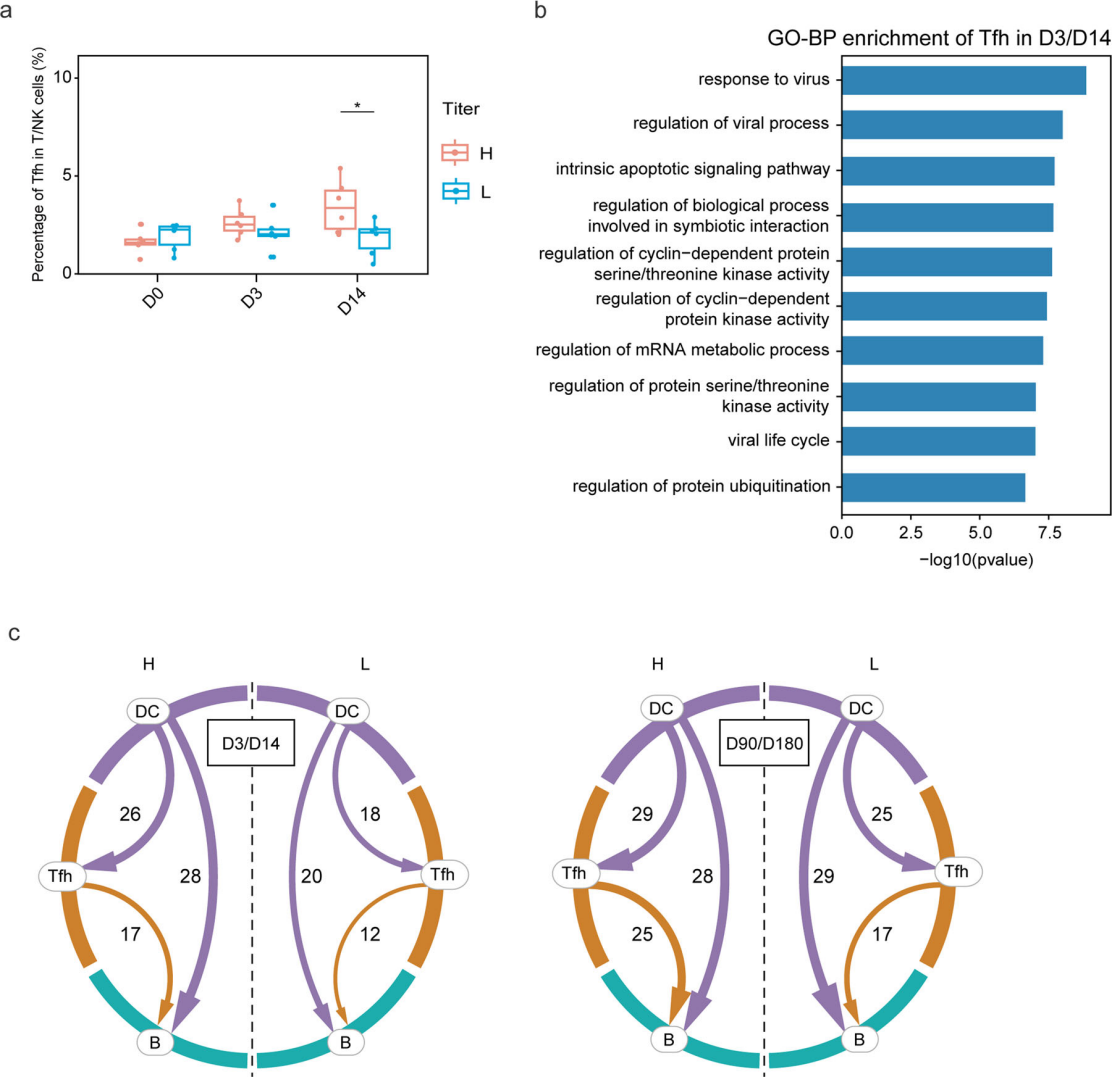
Tfh numbers are elevated in the high-antibody group. **a** Variation of the proportion of Tfh cell subtypes in T/NK cells at D0, D3, and D14. **b** The enriched GO-BP terms of the upregulated genes of Tfh in high-antibody-titer group at D3/D14 post-vaccination. *P* value was derived by Benjamini & Hochberg test. **c** The Circos plots showed the cell−cell interaction among cDCs, Tfh cells and B cells at D3/D14 and D90/D180. The width of each line represents the number of the protein−protein interactions involved in cell−cell communication.

In between cDCs and B cells, T cells play crucial bridging roles. After receiving antigen stimulation from cDCs, a portion of T cells differentiate to follicular helper T (Tfh) cells that aid B cell differentiation into plasma cells and memory cells at the germinal center. The proportions of Tfh cells in the high-antibody-titer group were significantly higher than those in the low-antibody-titer group at D14 (Fig. 6a). GO-BP enrichment analysis further showed that upregulated genes in Tfh cells in the high-antibody-titer group at D3/D14 were associated with virus response (Fig. 6b). The cell–cell communication analysis indicated that the context-dependent crosstalk between cDCs, Tfh cells and B cells was stronger in the high-antibody-titer group at D3/D14 and D90/D180 (Fig. 6c), suggesting that the biological processes underlying SARS-CoV-2 immunization, including antigen presentation and the activation of Tfh cells that help the maturation of B cells, were more active in the high-antibody-titer group.

### Energy metabolism fuels antibody production

**Fig. 7:**
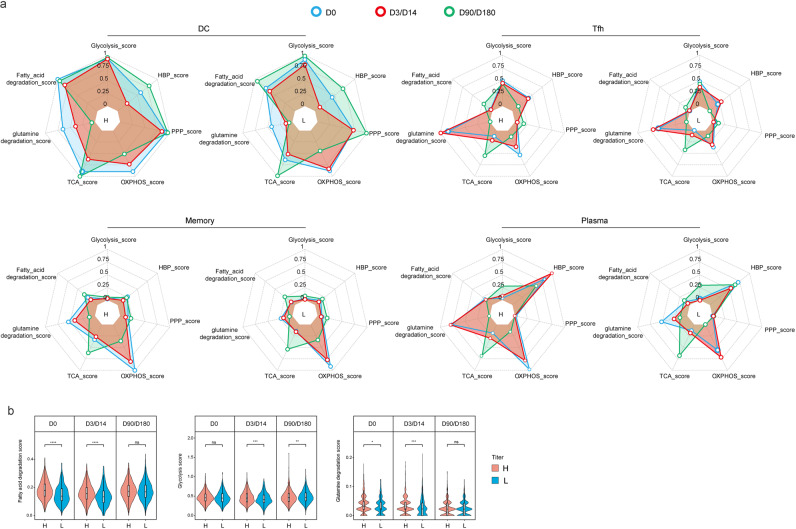
Energy metabolism of the key immune cells involved in humoral immunity. **a** Radar Plots shows energy metabolism pathway scores and relative carbon metabolism pathway scores normalized according to all scored cells, ranging from 0 to 1. **b** Violin plots show the Fatty acid degradation score, Glycolysis score and Glutamine degradation score of cDCs between high- and low-antibody-titer groups at D0, D3/D14 and D90/D180. Values are means ± SD. *P < 0.05, **P < 0.01, ***P < 0.001.

Due to the essential role of energy metabolism for all biological processes, we investigated energy metabolism dynamics of the immune cells involved in humoral responses, i.e., cDCs, Tfh cells, memory B cells and plasma cells (Fig. 7a; Supplementary Figs. S3, S4, S5, S6).

Mammalian cells can generate ATP molecules through either glycolysis in the cytosol or the tricarboxylic acid (TCA) cycle and oxidative phosphorylation (oxphos) in the mitochondria. Glycolysis relies solely on glucose while oxphos can utilize carbon sources from glucose, glutamine or fatty acids. cDCs showed the most active energy metabolism among all cell types, engaging both glycolysis and oxphos. Tfh cells also engaged both glycolysis and oxphos, though to a lower extent. Memory B cells and plasma cells, however, depended more on oxphos than glycolysis. In the high-antibody-titer group, the glutaminolysis pathway was upregulated in all four cell types. Meanwhile, in four cell types, the fatty acid oxidation pathway showed small but significant upregulation in the high-antibody-titer group. The glycolysis pathway showed relatively complex patterns. For instance, compared with that of the low-antibody-titer group, the glycolysis level of the high-antibody-titer group was comparable at D0, upregulated at D3/D14, and downregulated at D90/D180. Among all three major nutrients, glutamine appeared to be the most relevant to antibody levels.

The pentose phosphate pathway (PPP) is a metabolic pathway parallel to glycolysis that contributes to anabolism rather than catabolism. PPP was highly active in cDCs, while showing minimal activation in other cell types. When comparing the high- and low-antibody-titer groups, we found that the PPP of cDCs was more active at early time points in the high-antibody-titer group. The hexosamine biosynthetic pathway (HBP) is a branch of glycolysis that produces Uridine 5’-diphospho-*N*-acetylglucosamine (UDP-GlcNAc) for protein glycosylation^14^. Plasma cells engaged active HBP because glycosylation is critical for antibody activity. In contrast, memory B cells showed minimal HBP activity. The HBP activity of plasma cells also clearly correlated with antibody titers.

Since cDCs are metabolically active, we further analyzed their nutrient uptake pathways. Compared with the low-antibody-titer group, glucose and lipid uptake was generally upregulated in the high-antibody-titer group (Supplementary Fig. S7a, c). Consistently, glutamine uptake demonstrated an upregulation feature (Supplementary Fig. S7b). Particularly, cDCs at D0 displayed evident differences between the high- and low-antibody-titer groups, highlighting the importance of basal metabolic activity in fueling booster immunogenicity.

## Discussion

In this prospective study, we found that either homologous or heterologous third-dose booster vaccines are highly immunogenic for healthy adults, and the immune responses are durable for at least 6 months. The heterologous booster induces faster and more robust activation of plasma cells, leading to higher antibody production. Either homologous or heterologous booster vaccination can induce memory recall and de novo activation of B cells, while the induced antibody isotypes are substantially different between the two boosters. In both cases, the expanded B cell clones persist for months, explaining the sustained antibody levels observed in recipients. After regrouping the recipients into two groups according to their antibody titers, we found that the activation of cDCs and Tfh cells was positively correlated with the antibody titer. Active energy metabolism, especially glutaminolysis, was also crucial for antibody production, suggesting a potential strategy of nutrient supplementation for improving vaccine efficacy.

Our observation of sustained antibody levels at the 6th month time point suggested that the ZF2001 or BBIBP booster could both induce significantly increased and long-term humoral responses against COVID-19, similar to the phenomenon observed with mRNA vaccines^15^. Though the immune responses of the booster may wane over time, clinical observations have shown that the rate of confirmed infection, severity rate and mortality would remain low^16,17^. One possible reason for the more durable antibody titers post boosters compared to the two-dose vaccination strategy may be the increased SARS-CoV-2-specific T and B cells after booster injection. Previous studies have reported that COVID-19 vaccination could stimulate the spike- and RBD-specific memory B cells capable of continued protection against SARS-CoV-2^18,19^. Furthermore, booster vaccinations could lead to a rebound in immune response against SARS-CoV-2 variants compared to two-dose vaccination. The broad responses may be due to the co-evolution of B cells in response to different variants, including SHM and memory B cell clonal turnover^20,21^.

We further conducted single-cell profiling to reveal the underlying mechanisms of homologous and heterologous booster-induced early and long-term humoral immunity. We found that both the BBIBP-CorV/ZF2001 heterologous booster group and the BBIBP-CorV homologous booster group were able to recall functional B cell responses, induce de novo activation of naïve B cells and activate plasma cells (both IgA and IgG) and its differentiation of isotypes, possibly accounting for the significant increase in humoral responses post booster vaccination. This is in accordance with the fact that both IgG and IgA were highly produced antibody isotypes during serological and mucosal immunity, either post breakthrough infection or vaccination, in which IgG led to phagocytosis and cytokine secretion and IgA could further enhance IgG function and enrich cytokine secretion^22–24^. At D90/D180, we recorded persistence of expanded B cell clones for months, which led to the sustained antibody levels in the recipients. Our results showed that heterologous booster dose could induce a stronger and earlier antigen-specific immune response compared with homologous booster, possibly due to the differences in production strategy between these vaccines. BBIBP-CorV vaccine was developed based on 3 strains which covered the main population of SARS-CoV-2^25^, whereas ZF2001 targets the RBD of SARS-CoV-2 spike protein, containing a dimeric form of the RBD^26^. SARS-CoV-2 spike-specific memory B cells were enriched in convalescent patients several months post infection, while monoclonal antibodies against nucleoprotein (NP) and open reading frame 8 (ORF8) were non-neutralizing and non-protective in vivo, highlighting the significance of immunization-induced neutralizing spike-specific memory B cells^27^. Other heterologous vaccination regimens, such as the adenovirus-based ChAdOx1 (AstraZeneca) vaccine followed by mRNA BNT162b2 (Pfizer-BioNTech) vaccine for SARS-CoV-2, also generate robust immune responses that may provide better antibody responses than standard homologous ChAdOx1 vaccine series^28^. According to the results of antibody responses against the RBD in this study, it is not surprising that ZF2001 showed better performance than BBIBP-CorV^29^. However, BBIBP-CorV might induce more comprehensive cellular immunity than ZF2001, which remains to be further studied.

Enhancing humoral responses post vaccination could significantly improve vaccine efficacy. As such, exploring potential strategies to increase antibody titers is of great importance. Our study found that higher antibody titers were closely associated with activities of cDCs, which was first reported among SARS-CoV-2 research after the vaccines were approved for full use. DCs were the most important antigen-presenting cells that could activate downstream T cell and B cell immune responses, thus playing key roles in the pathogenesis of COVID-19^30,31^. SARS-CoV-2 infection is known to disrupt DC immune responses, leading to the possible stimulation of pro-inflammatory cytokines^32^. Although not reported in other SARS-CoV-2 vaccines, the function of DCs was previously demonstrated to influence the performance of modified vaccinia Ankara by cytokine production and activation of co-stimulatory molecules for T cell stimulation and cell death^33^. The development of DC-based therapeutic cancer vaccines have similarly revealed the vital role of DCs in vaccination^34^. This study revealed the importance of cDCs in increasing humoral immune responses post booster vaccination, suggesting the potential of activating Toll-like receptor and cytokine pathways to stimulate cDCs to elicit stronger T cell and B cell responses, with the added potential benefits of achieving higher antibody titers and even broader immunity against SARS-CoV-2 and its variants of concerns (VOCs).

Antibody production is highly dependent on energy supply. Optimizing energy metabolism thus represents another promising avenue to improve vaccine efficacy. This aspect has been sparsely studied prior to the development of the SARS-CoV-2 vaccines. Here, it was revealed that glutamine was the nutrient most relevant to antibody production. Glutamine is the most important nitrogen source in cells and plays a key role in amino acid transformation by transaminases^35,36^. Clinical studies have shown that glutamine supplementation can reduce the need for intensive care in COVID-19 patients^37^. Moreover, glutamine can promote both humoral and cellular immunity induced by influenza virus vaccine^38^. It is thus reasonable to hypothesize that glutamine supplementation during SARS-CoV-2 vaccination might have a benefit in promoting humoral immunity.

In summary, our study unveils the cellular mechanisms of booster-induced humoral immunity and points out several future directions to improve vaccine efficacy in the global battle against the quickly evolving SARS-CoV-2 viruses.

## Materials and methods

### Participant enrollment and sample collection

We conducted a prospective cohort study (NCT05095298) to evaluate the immunogenicity of the ZF2001 (PrSV group) or BBIBP-CorV (InaV group) booster injection primed with two doses of BBIBP-CorV vaccination. 71 and 63 participants were enrolled in the ZF2001 or BBIBP-CorV booster group, respectively, and neutralizing antibody titer was evaluated at D0, D3, D14, D90, and D180 post booster^39,40^. In this study, two participants from the BBIBP-CorV booster group were selected who had the top 25% neutralizing antibody titer (InaV H group), and two participants in the bottom 25% by D3 and D14 (InaV L group). In the ZF2001 booster group, four participants in the top 25% (PrSV H group) and four participants in the bottom 25% (PrSV L group) were enrolled. We further collected blood samples and conducted single-cell sequencing. This study was approved by the ethical committee of Huashan Hospital Affiliated to Fudan University (KY2021-749). Written informed consent was obtained from all the enrolled patients.

### Detection of anti-SARS-CoV-2 RBD neutralizing responses, antibody and IgG

We assessed the anti-RBD responses induced by a third dose of boosting vaccination, including plasma sVNT, anti-RBD antibody and IgG tests. Blood samples were taken from participants for serology tests at D0, D14 and D28 after the booster shot. Plasma sVNT titer was determined by using a SARS-CoV-2 Neutralizing Ab detection kit (PerkinElmer SuperFlex Anti-SARS-CoV-2 Neutralizing Ab Kit, SDX-57042). The anti-RBD antibody and IgG was measured by PerkinElmer SuperFlex Anti-SARS-CoV-2 Ab Kit and SuperFlex Anti-SARS-CoV-2 IgG Kit.

According to the manufacturer’s instructions (www.perkinelmer.com), we used superparamagnetic microparticles together with direct chemiluminescence technology to detect antibody in plasma samples. Plasma was serial diluted before detection, 50 μL diluted sample was added to each of the sample wells then mixed with 50 μL SARS-CoV-2 RBD protein labeled with acridinium ester. Signals were captured using PerkinElmer SuperFlex automatic chemiluminescence immunoassay analyzer.

To measure the neutralizing titer, the signals were converted to sVNT titer using a reference standard curve plotted with kit-suppled reagents. The sVNT titer was determined by the reciprocal of the last dilution that resulted in > 50% reduction of the chemiluminescence signal. The concentration of the anti-SARS-CoV-2 antibody or IgG of the samples was correlated with the luminous intensity. Written informed consent was obtained from each participant, and this study was approved by the Ethics Committee of Jiangsu Provincial Center of Disease Control and Prevention.

### pVNT

Blood samples were taken from participants at D0, D14, and D28 after the boosting vaccination. The samples were taken from the participants of the control groups at D0. Pseudovirus incorporated with spike protein from either prototype or variants (Alpha, Beta, Gamma, and Delta) were constructed using a procedure reported previously ^41^. On the day before transfection, 293 T cells were prepared and adjusted to the concentration of 5–7 × 10^5^ cell/mL with DMEM complete medium. 30 μg of plasmid pcDNA3.1.S2, which expressed the spike protein was transfected according to the previously established instructions. Afterwards, diluted G*∆G-VSV (VSV G pseudovirus) was added into flasks. Serial dilutions of human plasma and pseudoviruses with concentrations of 1300 TCID 50/mL were added into the plates. After incubation, HuH-7 cells were added into the plates. Chemiluminescence detection was performed after 24-h incubation. Serial fold of dilutions were prepared and the last column was used as the cell control without pseudovirus. Positive was determined to be ten-fold higher than the negative (cells only) in terms of relative luminescence unit (RLU) values. The Reed-Muench method was used to calculate the virus neutralization titer. The results were based on 3–5 replicates unless otherwise specified.

### Single cell collection, sorting, library preparation, and sequencing

PBMCs were isolated using HISTOPAQUE-1077 (Sigma-Aldrich, 10771) solution according to the manufacturer’s instructions. Briefly, 4 mL of fresh peripheral blood was collected in Ethylene Diamine Tetraacetic Acid (EDTA) anticoagulant tubes and subsequently layered onto HISTOPAQUE-1077. After centrifugation, PBMCs remained at the plasma-HISTOPAQUE-1077 interface and were carefully transferred to a new tube. Erythrocytes were removed using red blood cell lysis buffer and washed twice with sorting buffer (phosphate buffered solution (PBS) supplemented with 2% fetal bovine serum). The cell pellets were re-suspended in sorting buffer and were subsequently passed through a 40-µm Flowmi Cell Strainer.

The frozen PBMC cells were thawed according to the User Guide CG00039_Demonstrated Protocol Fresh Frozen Human PBMCs. Briefly, the frozen PBMC cells were thawed in a water bath at 37 °C and washed in warm complete growth medium (RPMI medium with 10% fetal bovine serum). The cell pellets were re-suspended in sorting buffer and were subsequently passed through a 40-µm Flowmi Cell Strainer. Single-cell suspensions were stained with 7-Aminoactinomycin D (7AAD) for fluorescence activated cell sorting (FACS), performed on a BD Melody instrument.

Cell viability and cell concentration were determined using a Countstar Automated Cell Counter. Cell viability of PBMCs was greater than 90% and the cell concentration was adjusted to 500–1200 cells/µL. PBMCs were loaded 18,000 cells/chip position using the 10× Chromium Next GEM Single Cell 5’ Kit v2. All the subsequent steps were performed following the standard manufacturer’s protocols. Purified libraries were analyzed by an Illumina nova-seq 6000 sequencer, with 150-bp paired-end reads.

### Processing of scRNA-seq data and quality control

Cell Ranger (version 5.0) was applied to filter low-quality reads, align reads to human reference genome (GRCh38), assign cell barcodes, and generate the UMI matrices. The output gene expression matrices were analyzed by R software (version 3.6.1) with the Seurat package (version 3.2.0). All samples were merged into one Seurat object using the *merge* function in Seurat. Low quality cells with < 200 genes detected, < 500 UMI counts detected or > 10% mitochondrial UMI counts detected were removed.

### Dimension reduction, unsupervised clustering and cell-type annotation

Dimension reduction and unsupervised clustering were performed according to the standard workflow in Seurat. *SCTransform* function was applied to normalize and find highly variable genes (HVGs) within the single-cell gene expression data. Mitochondrial genes, dissociation-induced genes and HLA genes were removed from HVGs for downstream analyses. Then, the effect of the percentage of mitochondrial gene counts was regressed out by using *SCTransform* function with parameter “vars.to.regress = ‘percent.mt’”. A principal component analysis (PCA) matrix was calculated to reduce noise by using *RunPCA* with default parameters. To remove batch effects from different samples, Harmony (version 1.0) was applied immediately after PCA with default parameters. Then UMAP and graph-based clustering were performed on the “harmony space” for visualization and cell clustering. The main immune cell types were annotated based on the expression pattern of DEGs and the well-known cellular markers from the literature. In the first-round of unsupervised clustering of all cells, we found that PTPRC and HBB were co-expressed in some clusters, so we removed these clusters for downstream analysis.

To identify subtypes within the B cell cluster, we performed a second-round of unsupervised clustering on B cells. The second-round clustering procedure was similar to the first-round clustering, which started from the expression matrix of the B cell subsets and then identified HVGs, calculated the PCA matrix, corrected batch effects by Harmony, detected cell clusters by Louvain algorithm and performed dimensionality reduction for visualization. The number of principal components was determined by the *Elbowplot* function in Seurat. DEGs were detected using the *FindAllMarkers* function with default parameters. Of note, we removed three clusters with high expression of CD3 or PPBP from the B cell subpopulations. We also noticed several clusters with a high percentage of dissociation-induced gene count in B cell subpopulations. The high expression of dissociation-induced genes might be artificially induced during the experiment. Therefore, we filtered out these clusters from our analysis.

### Differential expression analysis

To identify DEGs between two clusters, we used *FindMarkers* function to perform differential gene expression analysis. Genes with adjusted *P* < 0.05 were considered as DEGs.

### BCR analysis

Cell Ranger (version 5.0) was applied to align BCR-seq reads to the GRCh38 and to assemble BCR sequences. The preliminary BCR sequences were filtered to keep those characterized as high confident, full-length, productive. These were assigned with a valid cell barcode and an unambiguous chain type.

The Immcantation toolbox (version 4.3.0) was used for BCR downstream analysis. Immunoglobulin heavy chain (IgH) sequences were annotated using *AssignGenes.py* and *IgBLAST*. The SHazaM (version 1.1.0) package was used to evaluate sequence similarities based on their Hamming distance and estimate the distance threshold separating clonally related from unrelated sequences. BCR sequences were clustered into clonal groups using *DefineClones.py*, where the distance threshold was set to 0.2. *CreateGermlines.py* was then used to infer germline sequences for each clonal family and *observedMutations* was used to calculate SHM frequencies for each IgH sequence.

### Calculating gene scores

Gene expression score was calculated based on the sum of UMI counts of the genes in each gene set divide to total UMI counts detected in each cell.

### Cell–Cell interaction analysis

Cell–Cell interaction analysis was performed using CellPhoneDB (version 3.0)^42^ and only Protein–Protein interaction subsets were used. Width of the line represented the number of possibly activated protein couples among the linked cell types.

### Statistical analysis

The statistical tools, methods, and thresholds for each analysis are explicitly described with the results or detailed in the figure legends or the Materials and Methods section.

## Supplementary information

Supplementary Information

## Data Availability

All sequencing data have been uploaded to China National Center for Bioinformation (CNCB) Genome Sequence Archive (GSA) database under the accession number HRA002539. Custom scripts in this study are available upon request to the corresponding author.
