# Supplementary material for: Cellular basis of enhanced humoral immunity to SARS-CoV-2 upon homologous or heterologous booster vaccination analyzed by single-cell immune profiling
Source: Cell Discov. 2022 Oct 21;8:114. doi: 10.1038/s41421-022-00480-5 (PMC9587260; doi:10.1038/s41421-022-00480-5)
Supplement: Supplementary file 1 — Supplementary Information [file 41421_2022_480_MOESM1_ESM.pdf]

Supplementary Information for

Cellular basis of enhanced humoral immunity to SARS-CoV-2 upon homologous or heterologous booster vaccination analyzed by single-cell immune profiling

Author Information:

Jingwen Ai[1 2 *], Jingxin Guo[1 2 *], Haocheng Zhang[1 2 *], Yi Zhang[1 2 *], Haochen Yang[3 4 *], Ke Lin[1 2 *], Jieyu Song[1 2 *], Zhangfan Fu[1 2 *], Mingxiang Fan[5 *], Qiran Zhang[1 2], Hongyu Wang[1 2], Yuanhan Zhao[1 2], Zhangyufan He[1 2], An Cui[1 2], Yang Zhou[1 2], Jing Wu[1 2], Mingzhe Zhou[1 2], Guanmin Yuan[1 2], Boxi Kang[6], Ning Zhao[6], Yuanyuan Xu[1 2], Mengqi Zhu[1 2], Youhong Wang[1 2], Zemin Zhang[7], Ning Jiang[1 2 8 #], Chao Qiu[1 2 9 #], Chenqi Xu[3 10 11 #], Wenhong Zhang[1 2 12 13 #]

[1]National Medical Center for Infectious Diseases, Huashan Hospital, Fudan University, Shanghai, China

[2]Department of Infectious Diseases, Shanghai Key Laboratory of Infectious Diseases and Biosafety Emergency Response, Huashan Hospital, Fudan University, Shanghai, China

[3]State Key Laboratory of Molecular Biology, Shanghai Key Laboratory of Molecular Andrology, Shanghai Institute of Biochemistry and Cell Biology, Center for Excellence in Molecular Cell Science, Chinese Academy of Sciences, Shanghai, China

[4]University of Chinese Academy of Sciences, Beijing, China

[5]Department of Urology, Tongji Hospital, School of Medicine, Tongji University, Shanghai, China

[6]Analytical Biosciences Limited, Beijing, China

[7]Biomedical Pioneering Innovation Center (BIOPIC), School of Life Sciences, Peking University, Beijing, China

[8]State Key Laboratory of Genetic Engineering, School of Life Sciences, Fudan University, Shanghai, China

[9]Institutes of Biomedical Sciences, Fudan University, Shanghai, China

[10]School of Life Science and Technology, ShanghaiTech University, Shanghai, China

[11]Key Laboratory of Systems Health Science of Zhejiang Province, School of Life Science, Hangzhou Institute for Advanced Study, University of Chinese Academy of Sciences, Hangzhou, China

[12]National Clinical Research Center for Aging and Medicine, Huashan Hospital, Fudan University, Shanghai, China

[13]Huashen Institute of Microbes and Infections, Shanghai, China

[*]These authors contributed equally: Jingwen Ai, Jingxin Guo, Haocheng Zhang, Yi Zhang, Haocheng Yang, Ke Lin, Jieyu Song, Zhangfan Fu, Mingxiang Fan.

#**Correspondence:**
Prof. Ning Jiang (ningjiang@fudan.edu.cn), No. 12, Urumqi Middle Road, Jing'an District, Shanghai, 200040, China. Tel: 021-52887960
Prof. Chao Qiu (qiuchao@fudan.edu.cn), No. 12, Urumqi Middle Road, Jing'an District, Shanghai, 200040, China. Tel: 021-52887960
Prof. Chenqi Xu (cqxu@sibcb.ac.cn), No. 320, Yueyang Road, Xuhui District, Shanghai, 200031, China.
Prof. Wenhong Zhang (wenhongzhang_hs@126.com), No. 12, Urumqi Middle Road, Jing'an District, Shanghai, 200040, China. Tel: 021-52887960

Supplementary Table S1. Sample Information

SampleID	Antibody.titer	VaccineType	Age	Sex
118601	High	InaV	20	male
118701	High	InaV	26	male
117101	Low	InaV	40	male
119101	Low	InaV	29	male
113301	High	PrSV	25	female
115001	High	PrSV	38	male
100902	High	PrSV	32	female
120001	High	PrSV	36	female
119801	Low	PrSV	20	male
111902	Low	PrSV	42	male
112702	Low	PrSV	28	male
117801	Low	PrSV	27	female

Supplementary Table S2. The average of gene scores showed in Fig5a, 5b, 5c, Fig. 6b and Supplementary Figs3-6

**Fig. 5a**		
Average of Antigen Presentation score		
	H	L
mono	3.2039	3.5905
DC	9.4575	9.2845
pDC	4.9945	5.4102

**Fig. 5b**		
Average of Antigen Presentation score, cDC		
	H	L
D0	8.7270	7.5648
D3	9.2665	8.9269
D14	9.0741	8.7729
D90	10.7555	10.9458
D180	9.1336	9.0680

**Fig. 5c**		
cDC, D0		
	H	L
Toll−Like Receptor Score	0.1215	0.0714
TNF Biosynthesis Score	0.6580	0.4418
IFN Biosynthesis Score	0.2074	0.1350
IL−1 Regulation Score	0.1041	0.0855

Supplementary Table S2. The average of gene scores showed in Fig5a, 5b, 5c, Fig. 6b and Supplementary Figs3-6

**Fig. 6b**						
cDC						
	D0_H	D0_L	D3D14_H	D3D14_L	D90D180_H	D90D180_L
Fatty acid degradation score	0.1766	0.1374	0.1561	0.1321	0.1730	0.1684
Glycolysis score	0.4735	0.4635	0.4624	0.4234	0.4739	0.4845
Glutamine degradation score	0.0328	0.0240	0.0235	0.0229	0.0228	0.0228

**Fig. S3**						
cDC						
	D0_H	D0_L	D3D14_H	D3D14_L	D90D180_H	D90D180_L
TCA score	0.1428	0.1266	0.1192	0.1078	0.1579	0.1547
PPP score	0.2510	0.2094	0.2309	0.2024	0.2536	0.2637
Fatty acid degradation score	0.1766	0.1374	0.1561	0.1321	0.1730	0.1684
Glycolysis score	0.4735	0.4635	0.4624	0.4234	0.4739	0.4845
OXPHOS score	5.2601	5.1123	4.7875	5.1031	4.2413	4.1474
glutamine degradation score	0.0328	0.0240	0.0235	0.0229	0.0228	0.0228
HBP score	0.0000	0.0000	0.0000	0.0000	0.0211	0.0209

**Fig. S4**						
Tfh						
	D0_H	D0_L	D3D14_H	D3D14_L	D90D180_H	D90D180_L
TCA score	0.0755	0.0654	0.0877	0.0737	0.1189	0.1040
PPP score	0.0748	0.0738	0.0732	0.0716	0.0987	0.0930
Fatty acid degradation score	0.0579	0.0530	0.0579	0.0536	0.0772	0.0702
Glycolysis score	0.3172	0.3097	0.3037	0.2698	0.2997	0.2953
OXPHOS score	4.2105	3.5781	3.6010	3.4216	3.0493	2.9878
glutamine degradation score	0.0291	0.0277	0.0467	0.0268	0.0239	0.0240
HBP score	0.0000	0.0000	0.0000	0.0000	0.0000	0.0000

**Fig. S5**						
Memory B						
	D0_H	D0_L	D3D14_H	D3D14_L	D90D180_H	D90D180_L
TCA score	0.0954	0.0780	0.0931	0.0787	0.1199	0.1166
PPP score	0.0970	0.0847	0.0899	0.0788	0.1125	0.1007
Fatty acid degradation score	0.0803	0.0619	0.0723	0.0621	0.0949	0.0877
Glycolysis score	0.1562	0.1529	0.1588	0.1499	0.1656	0.1671
OXPHOS score	5.3992	5.2898	4.8750	4.8128	3.7603	3.7047
glutamine degradation score	0.0261	0.0248	0.0251	0.0249	0.0237	0.0237
HBP score	0.0000	0.0000	0.0000	0.0000	0.0000	0.0000

Fig. S6						
Plasma						
	D0_H	D0_L	D3D14_H	D3D14_L	D90D180_H	D90D180_L
TCA score	0.2454	0.2004	0.2175	0.2667	0.3693	0.3699
PPP score	0.1655	0.1549	0.1367	0.1829	0.1694	0.1844
Fatty acid degradation score	0.1929	0.2052	0.2059	0.1957	0.2420	0.2249
Glycolysis score	0.2664	0.2302	0.2476	0.2533	0.3121	0.3205
OXPHOS score	4.9222	4.9197	5.0604	4.9838	5.4015	4.9659
glutamine degradation score	0.0927	0.0845	0.0896	0.0831	0.0672	0.0626
HBP score	0.0434	0.0408	0.0482	0.0464	0.0486	0.0414

Fig. S1

a

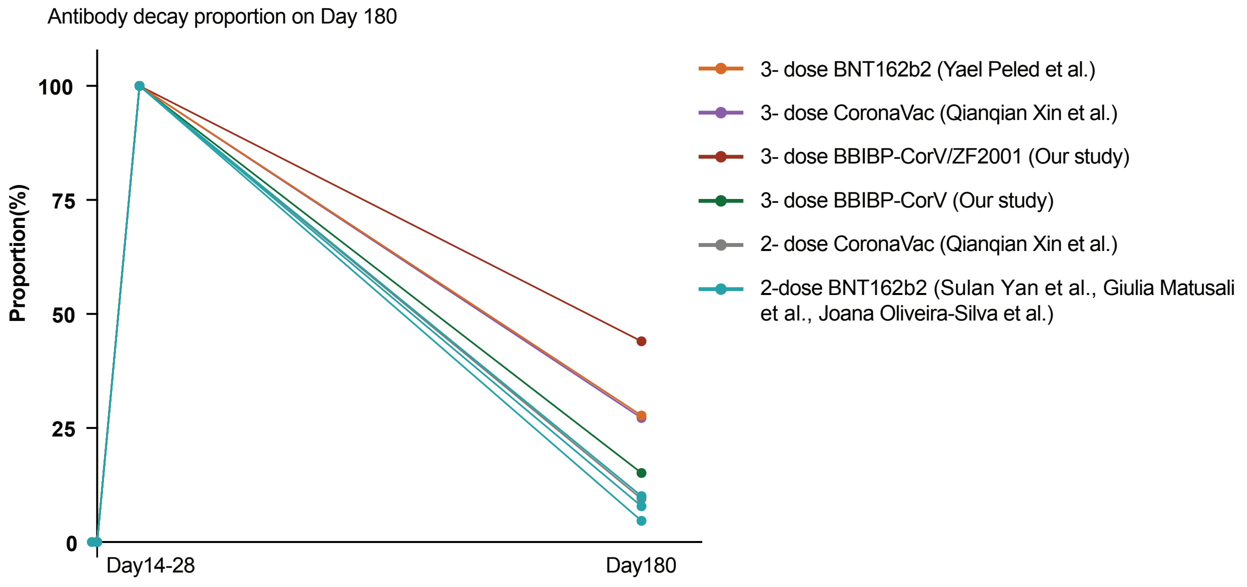

Antibody decay proportion on Day 180
Proportion(%)
100
75
50
25
0
Day14-28
Day180
3- dose BNT162b2 (Yael Peled et al.)
3- dose CoronaVac (Qianqian Xin et al.)
3- dose BBIBP-CorV/ZF2001 (Our study)
3- dose BBIBP-CorV (Our study)
2- dose CoronaVac (Qianqian Xin et al.)
2-dose BNT162b2 (Sulan Yan et al., Giulia Matusali et al., Joana Oliveira-Silva et al.)

b

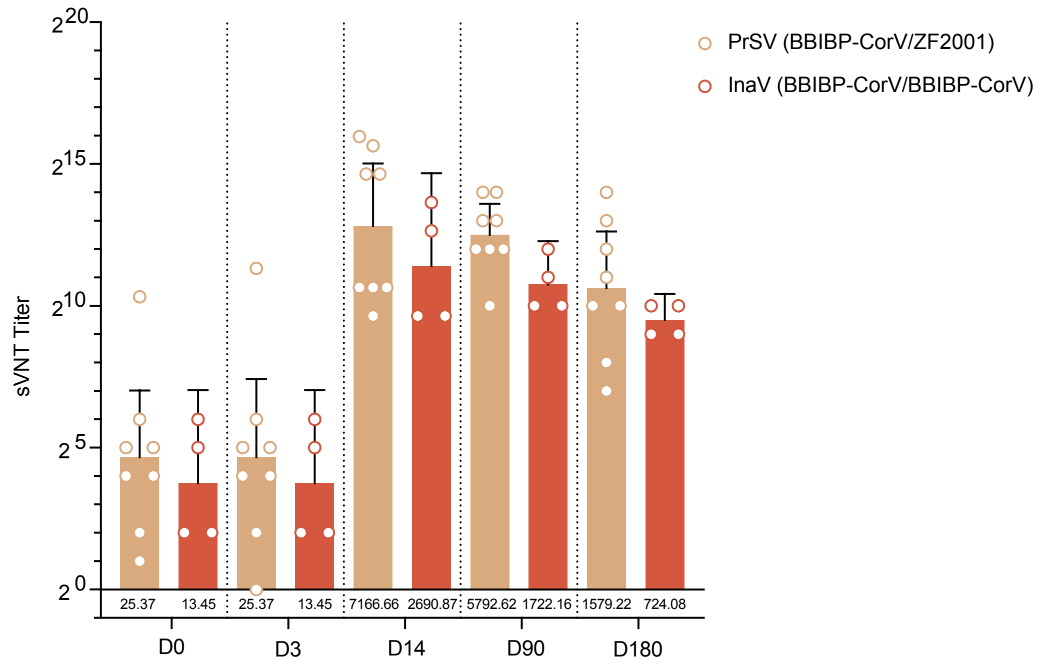

sVNT Titer
2 20
2 15
2 10
2 5
2 0
25.37
13.45
25.37
13.45
7166.66
2690.87
5792.62
1722.16
1579.22
724.08
D0
D3
D14
D90
D180
PrSV (BBIBP-CorV/ZF2001)
InaV (BBIBP-CorV/BBIBP-CorV)

Fig. S1 Comparison of antibody dynamics post vaccination of different boosters.

a. The antibody decline proportion compared to Day14/28 in different vaccination strategies (including 3-dose BNT162b2, 3-dose CoronaVac, 3-dose BBIBP-CorV/ZF2001, 3-dose BBIBP-CorV, 2-dose CoronaVac, and 2-dose BNT162b2). **b.** Humoral immune responses (sVNT) of each PBMC clusters at five time points (Day0, Day3, Day14, Day90, Day180) in BBIBP-CorV/ZF2001 heterologous booster group and BBIBP-CorV/BBIBP-CorV homologous booster group.

Fig. S2

a

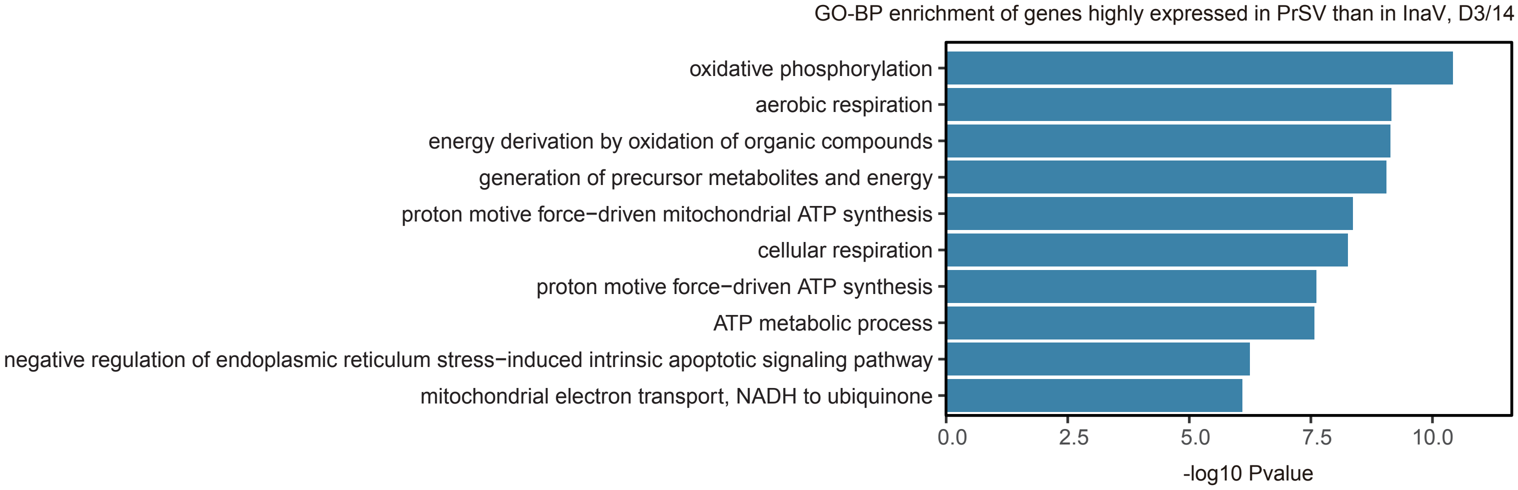
GO-BP enrichment of genes highly expressed in PrSV than in InaV, D3/14
oxidative phosphorylation
aerobic respiration
energy derivation by oxidation of organic compounds
generation of precursor metabolites and energy
proton motive force−driven mitochondrial ATP synthesis
cellular respiration
proton motive force−driven ATP synthesis
ATP metabolic process
negative regulation of endoplasmic reticulum stress−induced intrinsic apoptotic signaling pathway
mitochondrial electron transport, NADH to ubiquinone
0.0
2.5
5.0
7.5
10.0
-log10 Pvalue

b

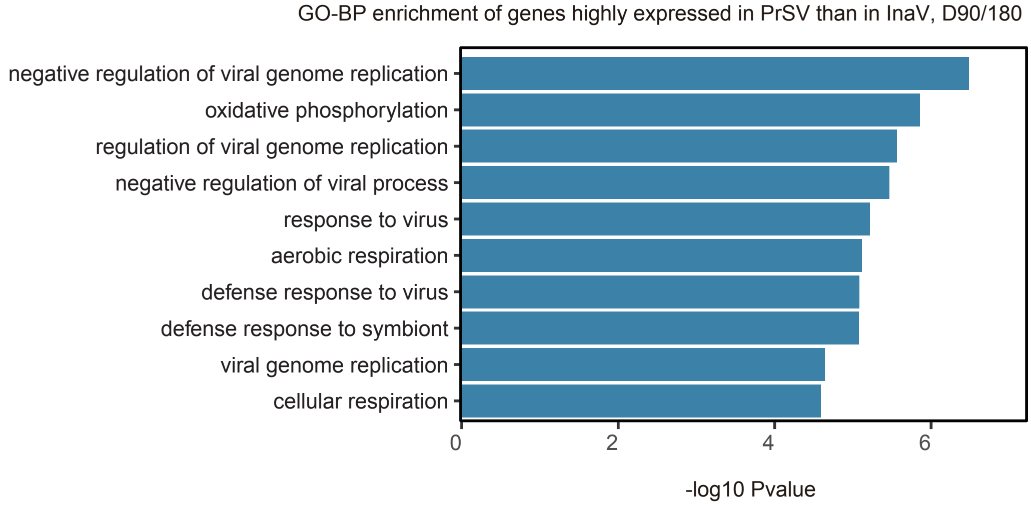
GO-BP enrichment of genes highly expressed in PrSV than in InaV, D90/180
negative regulation of viral genome replication
oxidative phosphorylation
regulation of viral genome replication
negative regulation of viral process
response to virus
aerobic respiration
defense response to virus
defense response to symbiont
viral genome replication
cellular respiration
0
2
4
6
-log10 Pvalue

Fig. S2 Enrichment analysis of differentially expressed genes between homologous and heterologous group.

a. The enriched GO-BP terms of up-regulated genes of plasma cells in InaV group at D3/D14 post-vaccination. P value was derived by Benjamini & Hochberg test.

b. The enriched GO-BP terms of up-regulated genes of plasma cells in InaV group at D90/D180 post-vaccination. P value was derived by Benjamini & Hochberg test.

Fig. S3

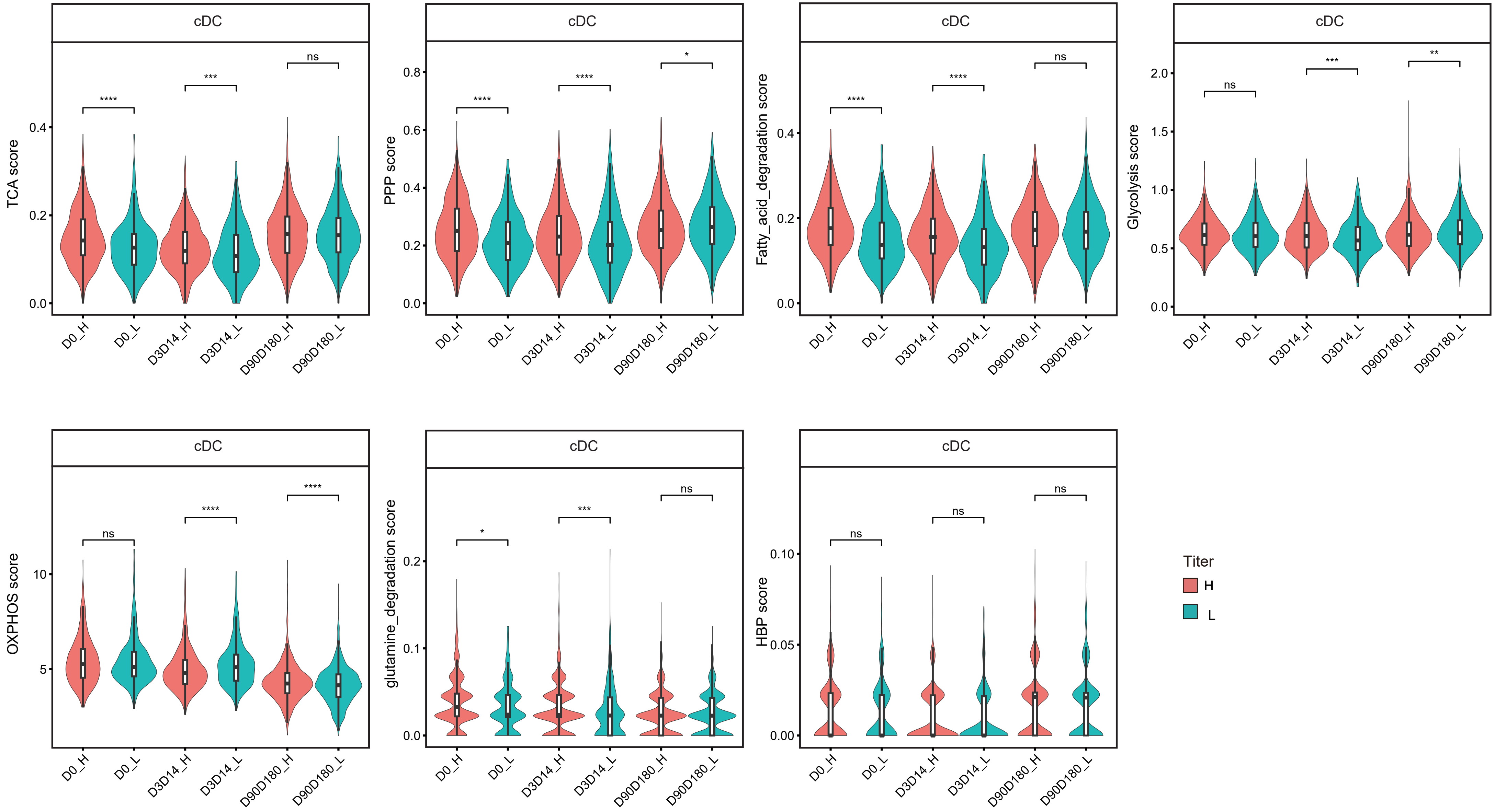
cDC
TCA score
PPP score
Fatty_acid_degradation score
Glycolysis score
OXPHOS score
glutamine_degradation score
HBP score
D0_H
D0_L
D3D14_H
D3D14_L
D90D180_H
D90D180_L
****
***
**
*
ns
Titer
H
L

Fig. S3 Energy metabolism of cDCs involved in humoral responses.

Vlnplots shows the energy metabolism pathway and relative carbon metabolism pathway scores of cDC between high- and low-antibody-titer groups at day 0, day 3/day 14 and day 90/day 180. Values are mean ± SD. *P < 0.05, **P < 0.01, ***P < 0.001. The average of gene scores were showed in Supplementary Table S2.

Fig. S4

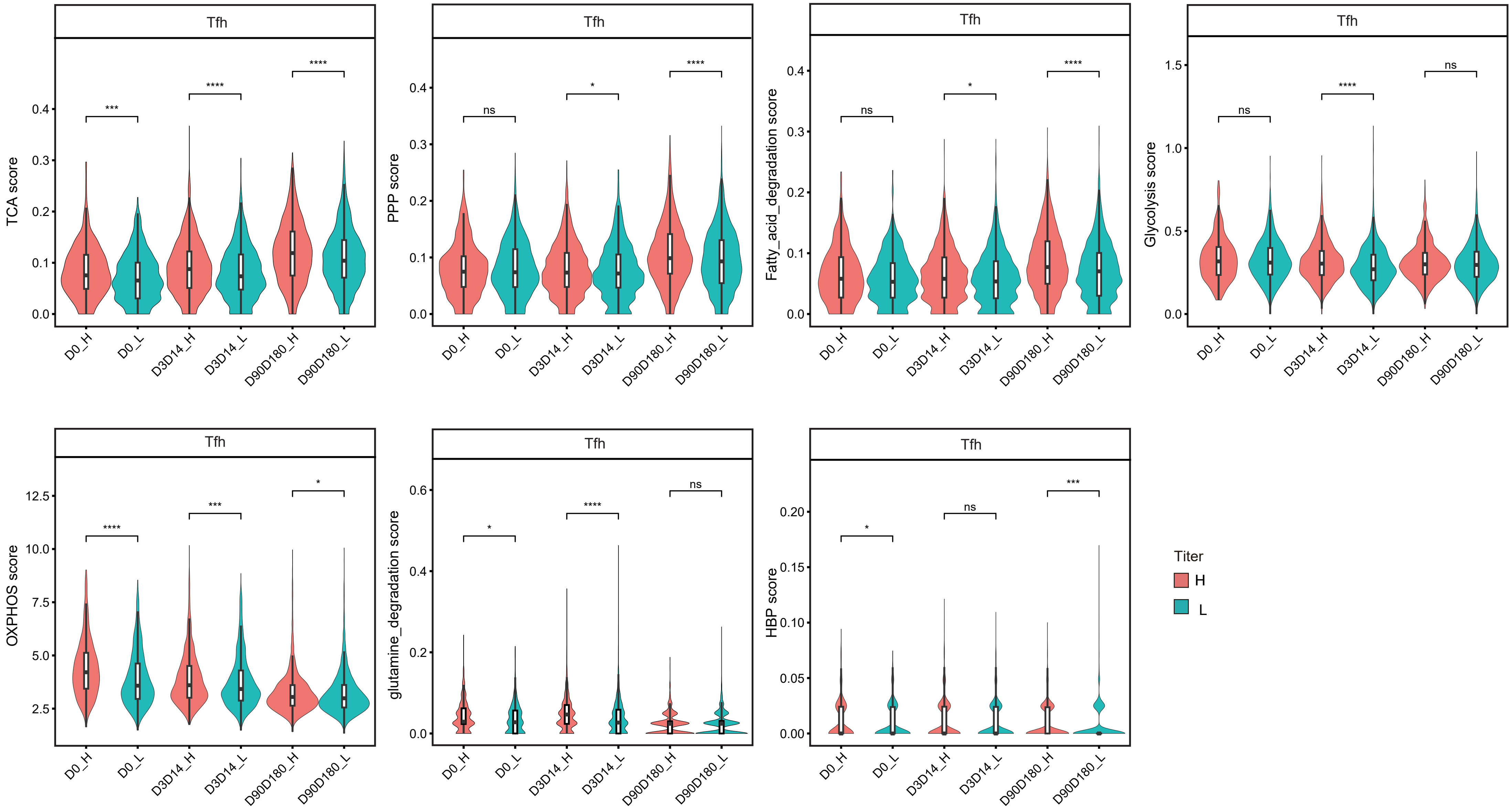
Tfh
TCA score
PPP score
Fatty_acid_degradation score
Glycolysis score
OXPHOS score
glutamine_degradation score
HBP score
D0_H
D0_L
D3D14_H
D3D14_L
D90D180_H
D90D180_L
Titer
H
L
ns
*
***
****

Fig. S4 Energy metabolism of Tfh cells involved in humoral responses.

Vlnplots shows the energy metabolism pathway and relative carbon metabolism pathway scores of Tfh between high- and low-antibody-titer groups at D0, D3/D14 and D90/D180. Values are mean ± SD. *P < 0.05, **P < 0.01, ***P < 0.001. The average of gene scores were showed in Supplementary Table S2.

Fig. S5

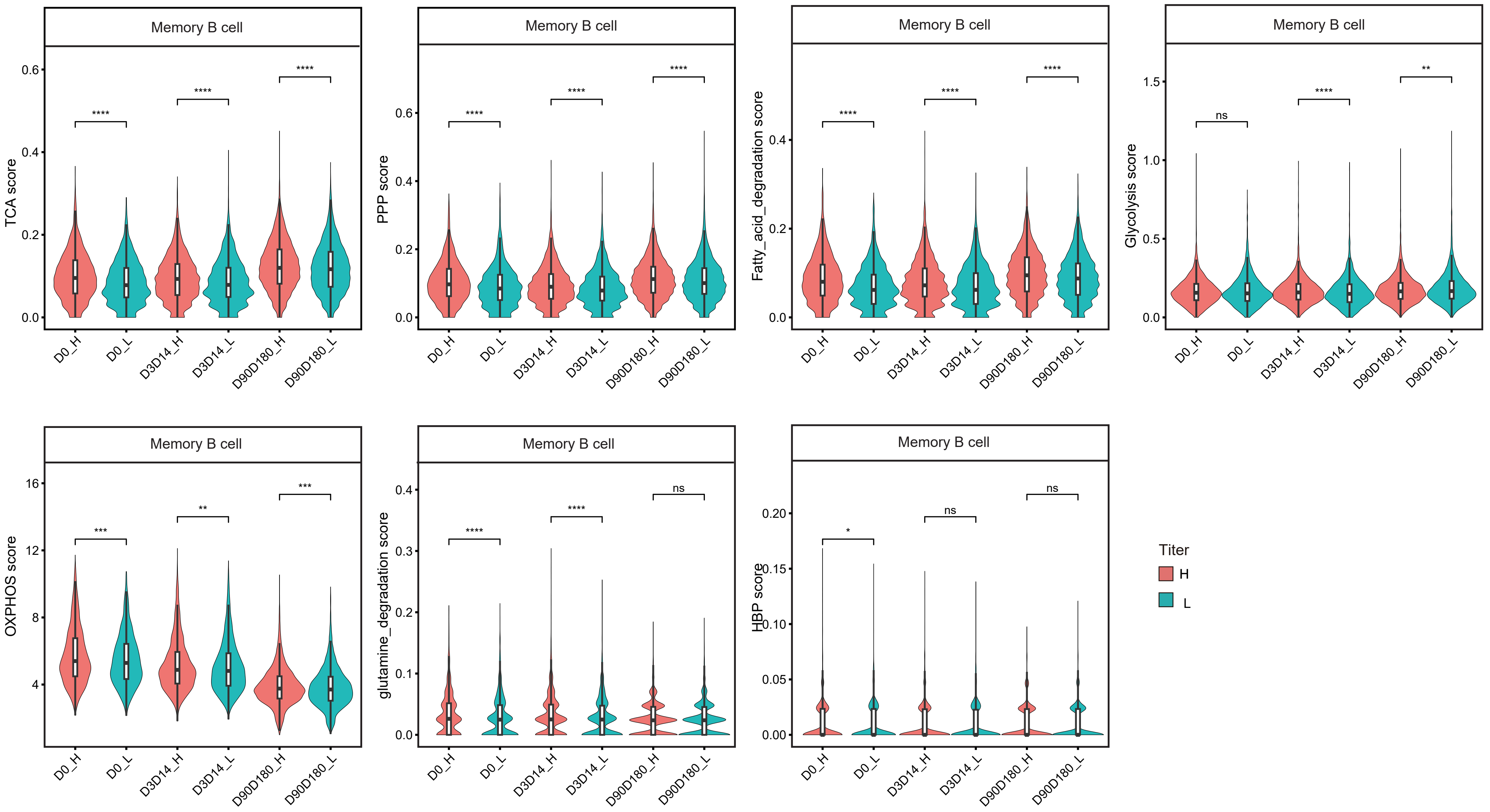
Memory B cell
TCA score
0.0
0.2
0.4
0.6
****
****
****
D0_H
D0_L
D3D14_H
D3D14_L
D90D180_H
D90D180_L
Memory B cell
PPP score
0.0
0.2
0.4
0.6
****
****
****
D0_H
D0_L
D3D14_H
D3D14_L
D90D180_H
D90D180_L
Memory B cell
Fatty_acid_degradation score
0.0
0.2
0.4
****
****
****
D0_H
D0_L
D3D14_H
D3D14_L
D90D180_H
D90D180_L
Memory B cell
Glycolysis score
0.0
0.5
1.0
1.5
ns
****
**
D0_H
D0_L
D3D14_H
D3D14_L
D90D180_H
D90D180_L
Memory B cell
OXPHOS score
4
8
12
16
***
**
***
D0_H
D0_L
D3D14_H
D3D14_L
D90D180_H
D90D180_L
Memory B cell
glutamine_degradation score
0.0
0.1
0.2
0.3
0.4
****
****
ns
D0_H
D0_L
D3D14_H
D3D14_L
D90D180_H
D90D180_L
Memory B cell
HBP score
0.00
0.05
0.10
0.15
0.20
*
ns
ns
D0_H
D0_L
D3D14_H
D3D14_L
D90D180_H
D90D180_L
Titer
H
L

Fig. S5 Energy metabolism of memory B cells involved in humoral responses.

Vlnplots shows the energy metabolism pathway and relative carbon metabolism pathway scores of memory B cell between high- and low-antibody-titer groups at D0, D3/D14 and D90/D180. Values are mean ± SD. *P < 0.05, **P < 0.01, ***P < 0.001. The average of gene scores were showed in Supplementary Table S2.

Fig. S6

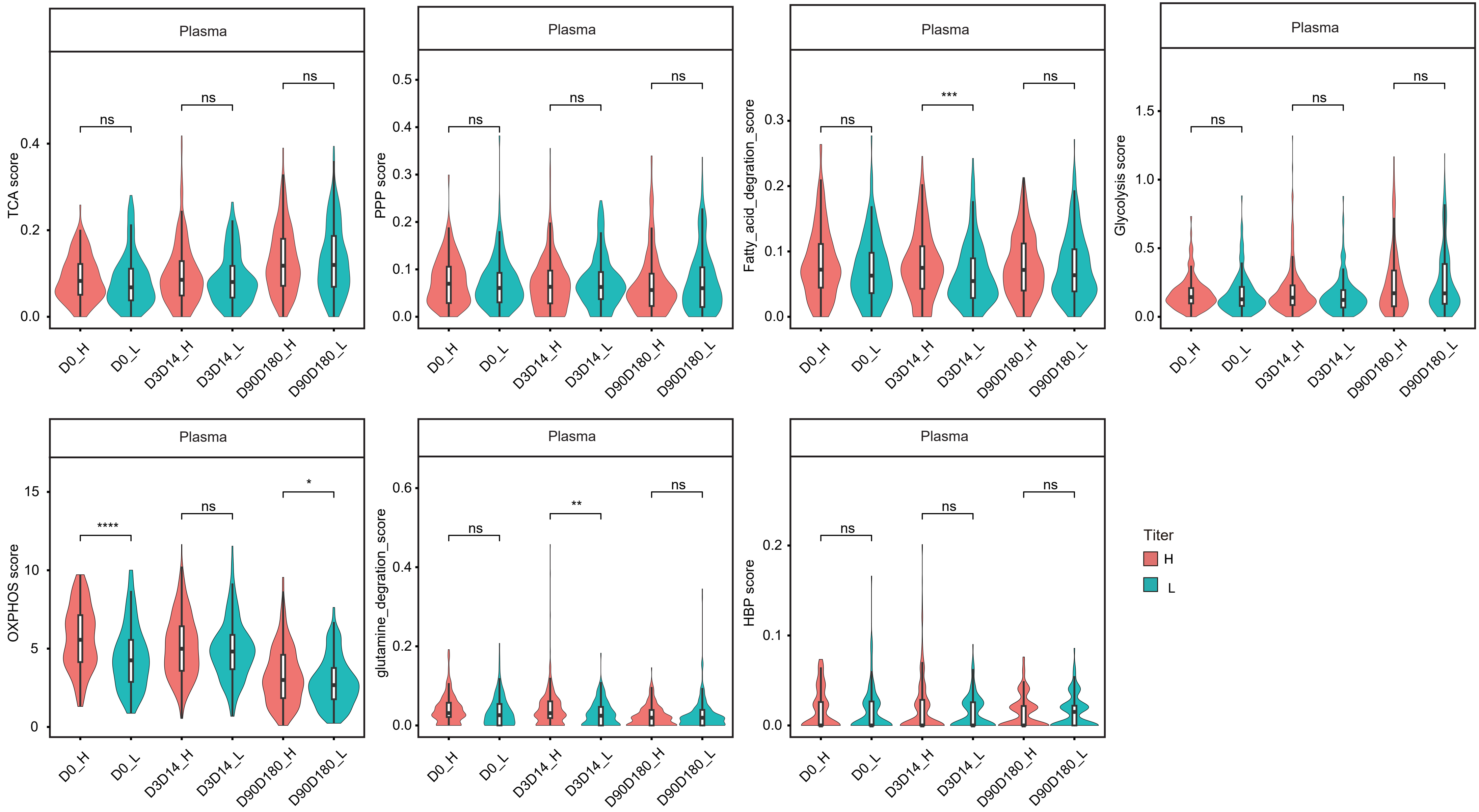
Plasma
TCA score
PPP score
Fatty_acid_degration_score
Glycolysis score
OXPHOS score
glutamine_degration_score
HBP score
ns
***
****
*
**
D0_H
D0_L
D3D14_H
D3D14_L
D90D180_H
D90D180_L
Titer
H
L

Fig. S6 Energy metabolism of plasma cells involved in humoral responses.

Vlnplots shows the energy metabolism pathway and relative carbon metabolism pathway scores of plasma between high- and low-antibody-titer groups at D0, D3/D14 and D90/D180. Values are mean ± SD. *$P < 0.05$, **$P < 0.01$, ***$P < 0.001$. The average of gene scores were showed in Supplementary Table S2.

Fig. S7

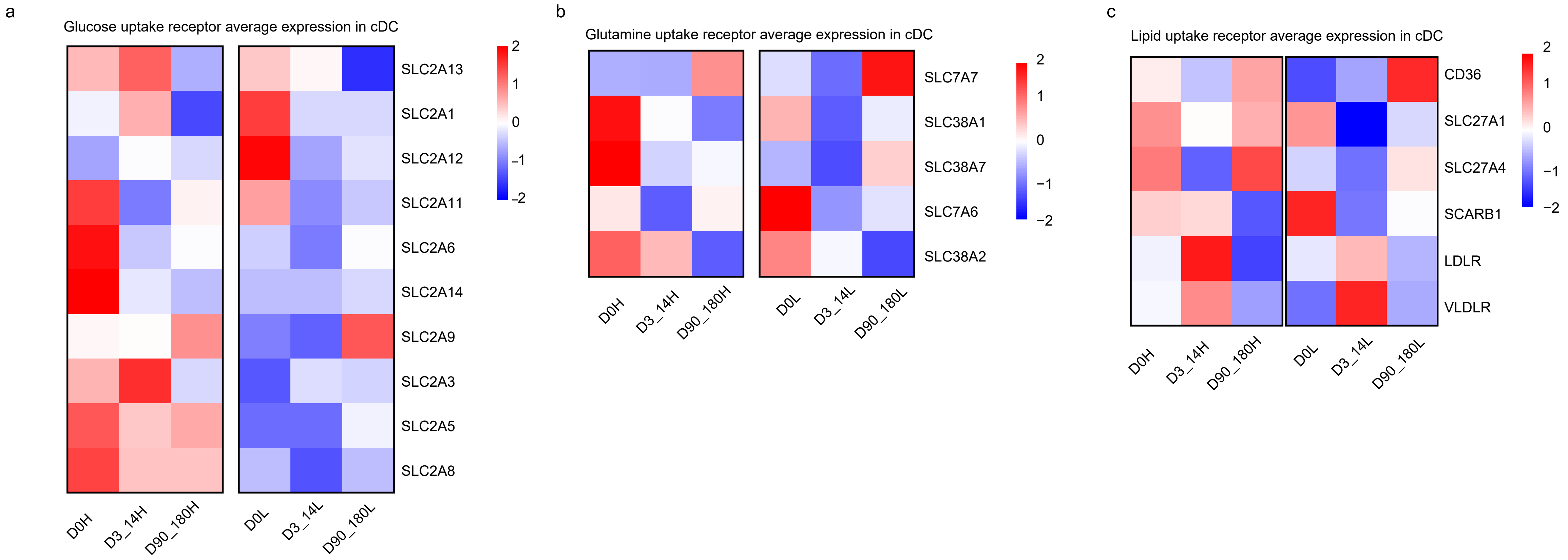
a
Glucose uptake receptor average expression in cDC
SLC2A13
SLC2A1
SLC2A12
SLC2A11
SLC2A6
SLC2A14
SLC2A9
SLC2A3
SLC2A5
SLC2A8
D0H
D3_14H
D90_180H
D0L
D3_14L
D90_180L
2
1
0
−1
−2
b
Glutamine uptake receptor average expression in cDC
SLC7A7
SLC38A1
SLC38A7
SLC7A6
SLC38A2
D0H
D3_14H
D90_180H
D0L
D3_14L
D90_180L
2
1
0
−1
−2
c
Lipid uptake receptor average expression in cDC
CD36
SLC27A1
SLC27A4
SCARB1
LDLR
VLDLR
D0H
D3_14H
D90_180H
D0L
D3_14L
D90_180L
2
1
0
−1
−2

Fig. S7 Nutrient uptake of conventional dendritic cells.

a. Heatmap of cDCs glucose uptake receptor average expression at D0, D3/D14 and D90/D180. **b.** Heatmap of cDCs glutamine influx receptor average expression at D0, D3/D14 and D90/D180. **c.** Heatmap of cDCs fatty acid uptake receptor average expression at D0, D3/D14 and D90/D180.
